# A placebo-controlled pilot study of intensification of antiretroviral therapy with mycophenolate mofetil

**DOI:** 10.1186/1742-6405-3-16

**Published:** 2006-05-26

**Authors:** Rupinderjeet Kaur, Roger Bedimo, Mary Beth Kvanli, Diana Turner, Leslie Shaw, David Margolis

**Affiliations:** 1 University of Texas Southwestern Medical Center at Dallas, Department of Medicine, Division of Infectious Diseases, Dallas, TX 75390, USA; 2 University of Pennsylvania, Philadelphia, PA 19104, USA; 3 North Texas Veterans Health Care Systems, Dallas, TX 75216, USA

## Abstract

**Purpose:**

We studied the safety, tolerability, virologic, and immunologic effects of mycophenolate mofetil (MMF) added to a stable antiretroviral therapy (ART) in the setting of low-level viremia.

**Methods:**

MMF 500 mg BID or placebo was given to patients thought to be adherent on stable ART with plasma viremia between 200 and 4000 copies/mL. At week 4 unblinding was performed and patients on placebo were offered open-label MMF.

**Results:**

Six patients were enrolled. At entry mean plasma HIV-1 RNA (VL) was 2.98 log_10 _copies/mL; mean CD4 count was 523. All subjects randomized to placebo elected to cross over to open label MMF. No significant adverse events were observed during MMF therapy. Three patients on MMF achieved VL < 50 copies/mL by week 4; a fourth had VL decline of > 0.5 log. Two patients on placebo had declines of VL. One of these had further decline on open label MMF. Cell surface markers of apoptosis, activation, and proliferation on CD4+ and CD8+ cells declined modestly or remained low. CD4 counts were stable at week 24. All but one subject had rebound of viremia by week 24, universally associated with missed doses of medication by pill count.

**Conclusion:**

MMF appears to be safe, and its administration lead to decreased T cell activation. During periods of adherence to therapy, the use of MMF was correlated with declines in viremia, but this small pilot study could not prove this association. Further study of MMF in patients with viremia should be considered for whom additional or alternative antiretrovirals are impractical.

## Introduction

The adjunctive use of inhibitors of nucleoside metabolism may exploit the reliance of HIV-1 on nucleoside pools for reverse transcription. Further, directly blunting host cell activation might have clinical benefits in HIV infection.

Mycophenolic acid (MPA) is a selective and reversible inhibitor of de novo synthesis of deoxyguanosine triphosphate (dGTP) [1,2]. MPA's effects are selective for lymphocytes, and it suppresses HIV replication through guanine depletion [3], increasing the efficacy of several reverse transcriptase inhibitors in vitro [4-6] and in vivo [7-10].

We hypothesized that MMF could improve virologic suppression in the setting of low-level viremia, preserving other antiretroviral agents for future use. We conducted a placebo-controlled pilot study to evaluate the safety, tolerability, and immunologic and virologic effects of the addition of MMF to an incompletely successful ART regimen. Volunteers with persistent viremia < 4000 but > 200 copies/ml were recruited. We found that MMF appears safe, and its use was associated with a decreased T cell activation as well as a short-term decline in plasma HIV-1 RNA. However, due to the confounding effect of non-adherence we could not irrefutably attribute the virologic effect seen to the activity of MMF.

## Methods

HIV-infected patients gave IRB-approved consent and were medically stable at study entry, without history of opportunistic infection within 12 months. All were on stable antiretroviral therapy including tenofovir, abacavir, and/or didanosine (agents shown to be potentiated by MMY in vitro; ref. 6) for ≥ 12 weeks with plasma HIV-1 RNA between 200 and 4000 copies/mL. Patients were carefully interviewed and felt to be adherent to therapy at entry by their long-term medical providers. Due to the theoretical possibility of clinical antagonism between thymidine analogs and MMF [4], patients receiving zidovudine or stavudine allowed to enroll if at least three of the following mutations had been detected in HIV-1 reverse transcriptase at a prior genotype: M41L, D67N, K70R, V75T, L210W, T215F/Y, K219E/Q, K65R, L74V, Q151M.

Patients with AIDS Clinical Trials Group (ACTG) grade IV liver function test abnormalities, grade III or higher renal insufficiency, grade III or higher leucopenia, or dementia thought to impair adherence were excluded. Study subjects were prohibited from concurrent use of systemic corticosteroids, hydroxyurea, or other immunosuppressive medications, cholestyramine, oral contraceptives, and probenecid or other inhibitors of tubular secretion.

Patients were first randomized to the addition of MMF 500 mg BID (Arm A) or matched placebo (Arm B) to their antiretroviral regimen (Step 1). Provider interviews and review of medication refill records were used to assess patient adherence. After 4 weeks of study therapy, unblinding was performed and patients on placebo offered open-label MMF for the remainder of the 24-week follow-up (Step 2), if they maintained HIV-1 RNA measurements of < 4000 copies/ml. Virologic and immunologic responses, MPA levels, and clinical status were monitored. Subjects on MMF during Step 1, regardless of their response to blinded MMF, were given the option of continued open-label MMF therapy and follow-up, or study discontinuation.

At each study visit, patients underwent clinical evaluation, HIV-1 RNA level by Roche Amplicor PCR assay, CD4 lymphocyte counts, hematology, and clinical chemistry (including serum lactate levels and anion gap analysis). Blood was also collected for cell surface marker studies.

Flow cytometry was performed on a FACS-Calibur, and data was analyzed with Cellquest software (Becton Dickinson, San Jose, CA) to measure expression of CD4, CD8, Annexin V, CD69, CD38, CD25 and Ki67 on CD4+ and CD8+ T cells. Annexin V-FITC Apoptosis Detection Kit I (BD Pharmingen) was used for detection of apoptosis. One million lymphocytes were examined from each study subject before the therapy was initiated, at weeks 4, 8 and 12 and at week 24 at the end of the therapy.

## Results

Six patients meeting the above criteria were enrolled. Baseline mean plasma HIV-1 RNA (VL) was 2.98 log_10 _copies/mL (range 1.9–3.9); and mean CD4 count was 523 (range 180–800). All subjects randomized to placebo elected to cross over to open label MMF. No significant adverse events were observed during MMF therapy. None of the patients experienced significant changes in blood hematocrit, metabolic profile, liver function tests or lipid profile during protocol therapy.

Four patients were randomized to receive MMF. Three of these achieved VL < 50 copies/mL by week 4, and elected to enter Step 2 of the study. One subject did not have a significant decline of plasma HIV-1 RNA on blinded MMF, and left the study after week 4. One patient on placebo had a significant decline of VL of > 0.5 log. This subject had a further VL decline of > 0.5 log copies/ml during Step II while receiving open-label MMF.

There was no significant change in mean CD4 count (422/mL at week 24) in subjects receiving MMF. All but one subject had rebound of viremia by week 24, universally associated with missed doses of medication by pill count. As observed in previous studies, average MPA AUC measured at week 4 was 19.40 (range 18.79–19.90) regardless of antiretroviral regimen [8,11].

The administration of low-dose MMF might decrease T cell activation, either by a direct immunomodulatory effect, or secondarily via an antiviral effect [7-10]. However, MMF has also been reported to induce apoptosis in the setting of HIV infection [10], although this effect may only be seen in activated cells [7,12]. Patient M4 received HAART and blinded MMF during Step 1 but did not display a virologic response. However, the Ki67, CD69, CD38 and CD25 levels declined while he was receiving MMF.

Subjects P1 and P2 received HAART and placebo during Step 1, and then HAART and open-label MMF during Step 2. These subjects had a decline in viral load during Step 1, presumably due to study-related improvements in adherence. While on open-label MMF, pill counts suggested non-adherence, correlated with a loss of virologic response. Levels of annexin and Ki67 decreased initially, but returned to baseline levels with the loss of virologic response. However, CD69 and CD25 levels declined somewhat and remained suppressed despite the loss of virologic response. In P1 the level of CD38 on CD8 cells also remained low despite viral rebound, whereas in P2 this marker increased after viral rebound.

Subject M1 displayed a gradual and persistent response to MMF during the course of the study. Annexin and Ki67 levels also declined during observation. However, activation markers increased at week 24. M3 and M2 received blinded MMF during the first 4 weeks of study, with declines in viremia. Annexin and Ki67 levels decreased initially, and surface levels of CD69, CD38 and CD25 remained low and stable. Both subjects lost virologic response; and non-adherence was simultaneously observed by pill count. During this time, while open-label MMF and HAART was prescribed but apparently taken irregularly, small but variable increases in Annexin and Ki67 were seen, as well as moderate increases in CD69, CD38 and CD25 levels in patient M2.

## Discussion

**Figure 1:**
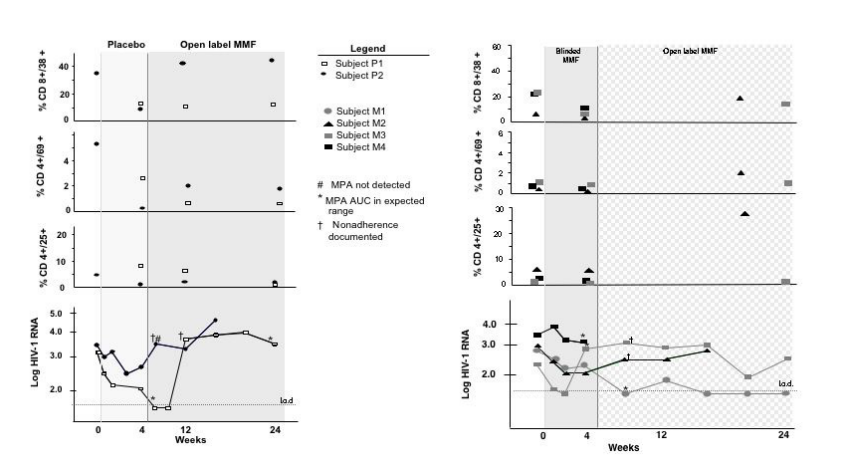
**Immunologic and virologic effects of MMF intensification**. Fig. 1A shows subjects initially assigned blinded placebo (light grey) who later elected to receive open-label MMF (grey). Fig. 1B shows those assigned blinded MMF (gray) who elected open-label MMF during weeks 4–24 (hatched grey). L.o.d.: limit of detection (< 50 HIV-1 RNA copies/ml).

In total therefore, MMF induced a persistent decrease in T cell activation in all but one patient (Fig 1A and 1B). Associated with suboptimal treatment adherence, virologic response was not durable in this patient population. The clinical scenario of partially successfully ART, similar to those screened for this study, is not uncommon. Treatment strategies for this group of patients are not well defined.

Continued therapy despite low-level viremia in the setting of drug resistance may be beneficial. Mutations conferring resistance to antiretroviral drugs commonly lower viral replicative capacity, and may blunt viremia In some treated patients [13,14]. Clinical and immunologic benefits can be maintained in patients with partial virologic suppression [15,16]. However, when partially effective treatment is continued, slow accumulation of resistance mutations may lead to increased viremia, and may jeopardize future treatment options. It is also reasonable to question whether continued exposure to the toxicity of multiple drugs is warranted in the face of limited virologic and immunologic response. A second approach in the face of partially effective antiretroviral therapy is the intensification of therapy. The risks of this approach include the development of resistance to the newly added antiretroviral(s), and cumulative toxicities.

A third option might be the use of an agent like MMF as a "stopgap" measure in patients without full virologic suppression who require antiviral therapy, but in whom active antivirals are not desired due to nonadherence, or not available due to drug resistance. Our data show a blunting of CD3+ cell activation in weeks 4 through 24, despite loss of virologic response in some patients. This, together with an absence in CD4 decline under MMF therapy is consistent with finding by other investigators in different settings [17-19].

It is possible that viral resistance to MMF might develop over time. One mechanism for this might be a shift in viral replication away from activagted lymphocytes and monocytes to cell types with lower levels of dependence on IMPDH type I, e.g. resting CD4+ T cells. However, substantial levels of viral replication in such cell populations. Alternatively, HIV RT could evolve higher affinity for native dGTP substrates. Three subjects responding to MMF initiated at the time of antiretroviral optimization during during our initial study [8] elected to extend MMF therapy under IRB oversight. Continued response to salvage therapy that including MMF, as measured by at least 0.5 log_10 _suppression of viral load and CD4 cell count stability, was observed for 27, 30, and 33 months, respectively prior to the clinical need for re-optimization of therapy. Only one patient developed a single new RT mutation during this time, although several changes in protease were observed.

In summary, in a short-term evaluation, MMF appears to be safe its use was associated with decreased T cell activation but the effect on VL suppression was not clearly ascertained, due to intermittent non-adherence to therapy during this study. Consistent with previous reports [7-10,17-19], we found no clinically significant cytopenias during MMF therapy. MMF has the potential to improve antiretroviral treatment response as well as delay virologic rebound. However, a comprehensive evaluation of the clinical efficacy of MMF will require a larger or a longer controlled study, due in part to the many factors which blunt treatment efficacy in patients with partially suppressed viremia.
